# Binding site density enables paralog-specific activity of SLM2 and Sam68 proteins in *Neurexin2* AS4 splicing control

**DOI:** 10.1093/nar/gkw1277

**Published:** 2016-12-19

**Authors:** Marina Danilenko, Caroline Dalgliesh, Vittoria Pagliarini, Chiara Naro, Ingrid Ehrmann, Mikael Feracci, Mahsa Kheirollahi-Chadegani, Alison Tyson-Capper, Gavin J Clowry, Philippe Fort, Cyril Dominguez, Claudio Sette, David J. Elliott

**Affiliations:** 1 Institute of Genetic Medicine, Newcastle University, Central Parkway, Newcastle NE1 3BZ, UK; 2 Department of Biomedicine and Prevention, University of Rome Tor Vergata, 00133 Rome, Italy; 3 Laboratory of Neuroembryology, Fondazione Santa Lucia, 00143 Rome, Italy; 4 Leicester Institute of Structural and Chemical Biology and Department of Molecular and Cell Biology, Henry Wellcome Building, University of Leicester, Lancaster Road, Leicester LE1 7RH, UK; 5 Institute for Cellular Medicine, Newcastle University, Framlington Place, Newcastle NE2 4HH, UK; 6 Institute of Neuroscience, Newcastle University, Framlington Place, Newcastle, UK; 7 Université Montpellier, UMR 5237, Centre de Recherche de Biologie cellulaire de Montpellier, CNRS, 34293 Montpellier, France

## Abstract

SLM2 and Sam68 are splicing regulator paralogs that usually overlap in function, yet only SLM2 and not Sam68 controls the *Neurexin2* AS4 exon important for brain function. Herein we find that SLM2 and Sam68 similarly bind to *Neurexin2* pre-mRNA, both within the mouse cortex and in vitro. Protein domain-swap experiments identify a region including the STAR domain that differentiates SLM2 and Sam68 activity in splicing target selection, and confirm that this is not established via the variant amino acids involved in RNA contact. However, far fewer SLM2 and Sam68 RNA binding sites flank the *Neurexin2* AS4 exon, compared with those flanking the *Neurexin1* and *Neurexin3* AS4 exons under joint control by both Sam68 and SLM2. Doubling binding site numbers switched paralog sensitivity, by placing the *Neurexin2* AS4 exon under joint splicing control by both Sam68 and SLM2. Our data support a model where the density of shared RNA binding sites around a target exon, rather than different paralog-specific protein–RNA binding sites, controls functional target specificity between SLM2 and Sam68 on the *Neurexin2* AS4 exon. Similar models might explain differential control by other splicing regulators within families of paralogs with indistinguishable RNA binding sites.

## INTRODUCTION

Around 95% of human genes encode multiple mRNA isoforms with different exon contents, thus greatly expanding the informational capacity of the genome. RNA binding proteins play a key role in creating different mRNA splice isoforms, by binding to target sites within pre-mRNAs and directing splice site choice by the spliceosome. These RNA–protein binding sites contribute to a splicing code through which the exon–intron structure of pre-mRNAs is deciphered (1–3). This splicing code includes RNA binding sites for proteins that activate and repress exon inclusion, called splicing enhancer and repressor sequences respectively, as well as the splice sites that they regulate. RNA binding sites can be both exon-proximal and sometimes deep intronic (4). In vertebrates, many splicing regulator proteins belong to families of evolutionarily related, similar sister proteins that often, but not always, regulate the same target exons. These sister proteins include the STAR proteins SLM2, Sam68 and SLM1; the Transformer proteins Tra2α and Tra2β; the Polypyrimidine Tract Binding Proteins PTBP1-3; the Muscleblind proteins MBNL1–3; the Epithelial Specific RNA Splicing regulator proteins ESRP1 and ESRP2; RNA binding Fox1 homolog proteins RBFOX1–3; TIAL and TIA1; as well as others (5). Many of these paralogs were created by gene duplications very early in vertebrate evolution. However, to what extent very similar splicing regulator paralogs might select overlapping versus distinct targets is often poorly understood, as are why such similar paralogs have been maintained often over considerable periods of evolutionary time.

An important example of differential splicing regulator paralog function is found within the nervous system. Here, the STAR family RNA binding proteins Sam68 (also known as KHDRBS1), SLM1 (also known as KHDRBS2) and SLM2 (also known as KHDRBS3 and T-STAR) differentially regulate splicing patterns of the *Neurexin1–3* genes involved in brain function (6–9). The *Neurexin1–3* genes encode trans-membrane pre-synaptic proteins, mutations in which are associated with various conditions including schizophrenia and autism (10), indicating the crucial role played by their regulation for proper brain functions. Each *Neurexin* gene has several alternative exons called alternative segments 1–5 (abbreviated AS1–AS5), and through alternative splicing of these exons can produce many different mRNA isoforms (11). While SLM2 and Sam68 usually function interchangeably in splicing regulation within transfected cells and both repress inclusion of the *Neurexin1* and *Neurexin3* AS4 exons, only SLM2 but not Sam68 regulates the *Neurexin2* AS4 exon (6). These AS4 splicing patterns are physiologically important, as manipulation of *Neurexin1* and *Neurexin3* AS4 alternative splicing affects both mouse behavior and synapse function (12,13).

SLM2 and Sam68 are 62% identical, and have similar modular organisations, including a STAR domain comprising a KH domain and flanking regions that mediate protein–protein and protein–RNA interactions, an RG (arginine/glycine)-rich region and C-termini enriched in tyrosine residues (14). Based on atomic level resolution from X-ray crystallography and nuclear magnetic resonance (15), SLM2 and Sam68 proteins bind RNA via their STAR domains, with largely overlapping but not identical RNA–protein contacts with the same U(A/U)AA target sequence (abbreviated UWAA) (15–17). There is a 51-nucleotide cluster of intronic Sam68/SLM2 binding sites immediately downstream of the *Neurexin2* AS4 exon that mediates the splicing response to SLM2 (6), but the features underlying differential regulation of *Neurexin2* AS4 splicing by SLM2 and not by Sam68 remain unclear. Herein, we have investigated the features of the SLM2 and Sam68 proteins and of the target *Neurexin2* pre-mRNA that enable this different splicing activity, and elucidate distinct physiological properties of these closely related protein paralogs.

## MATERIALS AND METHODS

### Crosslinking-immunoprecipitation experiment

The CLIP assay was performed as previously described (18,19). In brief, dissociated cortex tissues were irradiated on ice (100 mJ/cm^2^). The cell suspension was centrifuged at 4000 rpm for 3 min, and the pellet was incubated for 10 min on ice in lysis buffer (50-mM Tris, pH 8.0, 100-mM NaCl, 1% NP-40, 1-mM MgCl_2_, 0.1-mM CaCl_2_, 0.5 mM Na_3_VO_4_, 1-mM DTT, protease inhibitor cocktail [Sigma-Aldrich] and RNase inhibitor [Promega]). Samples were briefly sonicated and incubated with DNase (RNase-free; Ambion) for 10 min at 37°C and then centrifuged at 15 000 g for 10 min at 4°C. One milligram of extract was immunoprecipitated using anti-STAR (Santa Cruz Biotechnology, Inc.) or IgG (negative control) in the presence of protein G magnetic (Life Technologies). 1 U RNase I (Ambion) was added to immunoprecipitates and incubated for 2 h at 4°C under rotation. After stringent washes, 10% was kept as a control to test efficiency of immunoprecipitation by western blot, while the rest of the immunoprecipitated samples were treated with 50 μg Proteinase K and incubated for 1 h at 55°C. RNA was then isolated by standard procedures and retrotranscribed with random primers, using M-MLV reverse transcriptase (Promega). qPCR was performed using LightCycler 480 SYBR green I Master and the LightCycler 480 System (Roche) according to the manufacturer's instructions. RNA associated with STAR proteins is represented as fold enrichment relative to IgG samples. All primers used are listed in Supplementary file 1.

### Fluorescence polarization

Sam68 and SLM2 STAR domains were produced as previously described (15). RNA oligonucleotides were purchased from Dharmacon, GE Healthcare, deprotected according to the manufacturer's instructions, lyophilized, and resuspended in ddH_2_O. All RNAs used for fluorescence polarization contained a fluorescein tag and three cytosines at the 5΄ end of the sequence described in Table 1.

**Table 1.** Affinity measurements of the STAR domains of T-STAR and SAM68 proteins to target RNAs from *Neurexin2* and *Stxbp5l* carried out using fluorescence polarization (FP)

	SLM2	SAM68
Nrxn2 WT	5.9 ± 0.9	2.9 ± 0.3
Nrxn2 mutant 7	3.9 ± 0.4	3.4 ± 0.3
UAAAAx4	7.9 ± 1.2	6.9 ± 0.8
Stxbp5l 1	24.0 ± 5.9	15.5 ± 4.5
Stxbp5l 2	18.2 ± 3.3	13.8 ± 2.6
Poly(C)	>100	>100

Poly(C) was used as a negative control. Affinities are given in *K*_d_ (μM) with standard deviations derived from three independent experiments. The RNA molecules used for FP experiments were Nrxn2 WT:

5΄-AAUUAAUUAAUUAAUUAACUAACUAACUAACUUUAAAAACACGAUCUUAAA-3΄;

Nrxn2mutant7:

5΄-AAUUAAUUAAUUAAUUAACUAAC**CC**AC**CC**AC**CC**UAAAAACACGAUCUUAAA-3΄;

UAAAAx4: 5΄-UAAAAUAAAAUAAAAUAAAA-3΄;

Stxbp5l 1: 5΄-ACAGUUUAAAAUUUGAUAAAAUUU-3΄;

Stxbp5l 2: 5΄-UUACAUUUAAAAGAUGAUUUAAAAA-3΄.

Poly(C): 5΄-CCCCCCCCC-3΄.

Fluorescence polarization experiments were carried out in black 96-well plates with a 50 μl sample volume per well in 10 mM Tris pH 7, 100 mM NaCl, 0.1% β-mercaptoethanol. Sam68 and SLM2 domains were serially diluted across the plate from 200 to 0 μM. Fluorescein-labeled RNA was then added at 0.2 μM final concentration. Plates were analyzed using a Perkin Elmer Victor X5 plate reader at excitation wavelength of 531 nm and emission at 595 nm, and experiments were carried out in triplicate.

### Genomic sequences

*Neurexin1–3* sequences were retrieved from NCBI database (http://www.ncbi.nlm.nih.gov), using the annotation search tools available in the Geneious 7.1.5 package (Biomatters, http://www.geneious.com/). Accession numbers: *Homo sapiens* (NG_011878.1; NC_000011.10; NC_000014.9), *Mus musculus* (18189; 18190; 18191), *Sarcophilus harrisii* (100090577; 100917908; 100928491), *Macropus eugenii* (ENSMEUG00000007055; ENSMEUG00000016641; ENSMEUG00000001347), *Ornithorhynchus anatinus* (100090577; 100073756), *Pelodiscus siniensis* (102449503; 102447445; 102449503), *Alligator mississippiensis* (102558540; 102566256; 102570703), *Xenopus tropicalis* (100127647), *Danio rerio* (BX255907; CR751231; BX571723; NW_001884422).

### Minigenes and creation of hybrid proteins

Minigenes were cloned into pXJ41 using the primers in Supplementary File S1, and mutagenesis was carried out by overlap PCR as previously described (20). Minigene splicing patterns were analysed in HEK293 cells 24 h after co-transfection either with GFP, SLM2-GFP or SAM68-GFP as previously described (6). Tissue culture, transfection of HEK293 cells, RNA purification and RT-PCR was carried out using primers and conditions as previously described (21) and the RT-PCR products were analysed by capillary gel electrophoresis (20). To enable analysis of splicing patterns as well as monitoring the expression of transfected splicing factors, transfected cell cultures were split into two portions and analysed by RT-PCR and western blotting as previously described (22). Splicing patterns were measured as percentage splicing exclusion (6). All primers used for splicing assays are provided in the supplementary information. Hybrid SLM2 and Sam68 proteins were made by G block synthesis, and cloned in frame with GFP.

### Detection of gene expression in mouse brain tissue

RNA was prepared from brain tissue dissected from wild type and *Khdrbs3* (SLM2) and *Khdrbs1* (SAM68) (23) knockout mice using TRIzol (Life Technologies). The cDNA was generated using Superscript 3 (Invitrogen) and analysed by RT-PCR using primer pairs described in the Supplementary Information.

### Statistical analysis

Bar charts were plotted and statistical analyses performed using Graphpad Prism (Graphpad software).

### Western blots

Western blots were carried out as described previously (21). Proteins were detected by western blotting using antibodies specific for GFP (Clontech 632 381 Living Colors A v monoclonal antibody); SLM2 (6) SAM68 (Santa Cruz anti-Sam68 sc-333) and for actin β-Actin (Sigma-Aldrich, A5441,1:2000 dilution).

## RESULTS

### SLM2 and SAM68 proteins bind indistinguishably to the *Neurexin2* pre-mRNA

**Figure 1.**
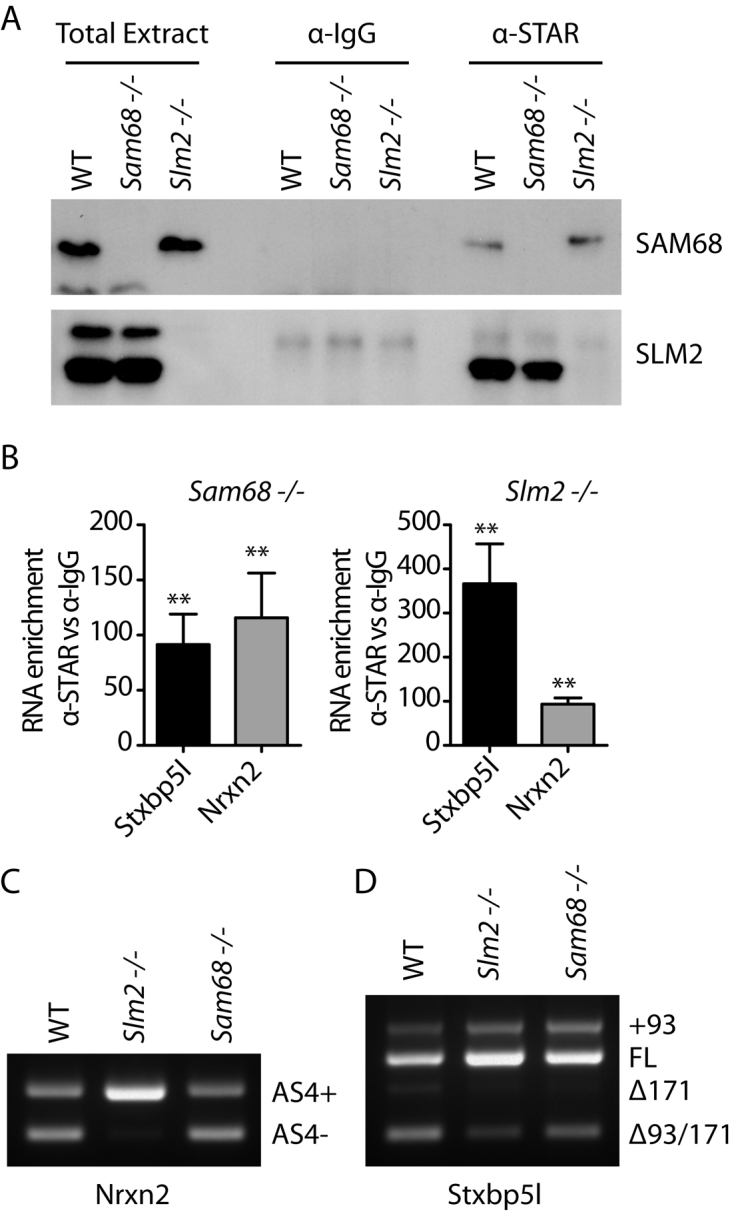
Splicing patterns and RNA protein interactions of the *Neurexin2* and *Stxbp5l* pre-mRNAs within the mouse cortex. (**A**) Western blot analysis of CLIP experiments for SLM2 and SAM68 proteins. Immunoprecipitations were carried out using an anti-STAR antibody in wild-type (WT), SAM68 (*Sam68^−/−^)* or SLM2 (*Slm2^−/−^)* null mouse cortex, and probed with an antibody that can detect both SLM2 and Sam68. (**B**) qPCR analysis of SLM2 and SAM68 protein binding to the *Stxbp5l* and *Neurexin2* pre-mRNAs assayed by CLIP experiments using an anti-STAR antibody in *Sam68 ^−/−^* or *Slm2^−/−^* null mouse cortex, respectively. Data shown in the bar charts are averages from four biological replicates. Error bars show standard error of the mean. Statistical significance of mRNA enrichment of STAR antibody with respect to control IgG was addressed using t tests, and is shown on the bar charts as: **P < 0.01. (C, D) Endpoint RT-PCR and agarose gel electrophoresis analysis of the endogenous splicing patterns of *Neurexin2* AS4 (**C**) and *Stxbp5l* genes (**D**) in WT, *Sam68^−/−^* and *Slm2^−/−^* mouse cortex.

The mouse cortex is a brain structure that expresses both Sam68 and SLM2 but negligible levels of SLM1 (8). To monitor binding of endogenous SLM2 and Sam68 proteins to the *Neurexin2* pre-mRNAs within the mouse cortex we used CLIP (Cross Linking Immunoprecipitation), with an antibody that recognizes both Sam68 and SLM2 (anti-STAR antibody, Figure 1A). To properly discriminate between the two proteins, CLIP experiments were performed to detect SLM2 in a *Sam68* null mouse background, and to detect Sam68 in a *Slm2* null mouse background.

Western blot analysis showed efficient immunoprecipitation of both Sam68 and SLM2 from the mouse cortex (Figure 1A), whereas SLM1 was not detected in either background (data not shown). Quantitative analysis of the immunoprecipitates showed efficient SLM2 protein cross-linking to the *Neurexin2* pre-mRNA (Figure 1B; ∼100-fold enrichment of *Neurexin2* precipitation with SLM2 compared with IgG), in line with the strong effect of SLM2 expression on AS4 splicing in the cortex (Figure 1C). Surprisingly however since Sam68 does not control *Neurexin2* AS4 exon skipping within the mouse cortex (Figure 1C), Sam68 protein was also efficiently cross-linked to the *Neurexin2* pre-mRNA (Figure 1B; ∼100-fold enrichment of *Neurexin2* precipitation with Sam68 compared with IgG). Precise measurements using fluorescence polarization (FP) also showed that SLM2 and Sam68 have very similar *in vitro* affinity for the 51 nucleotide UWAA-rich sequence downstream of the *Neurexin2* AS4 exon (Table 1). Hence, these data indicate that the different splicing activity of SLM2 and Sam68 on *Neurexin2* AS4 does not correlate with detectable differences in Sam68 and SLM2 protein-binding levels to the endogenous pre-mRNA.

To test whether similar joint binding also occurred on another functional SLM2 target in the mouse brain, we analyzed a 171 nucleotide exon within the *Stxbp5l* gene, which is one of the few other known splicing targets of SLM2 besides the *Neurexin1–3* genes (6,13). CLIP also detected binding of both Sam68 and SLM2 to the *Stxbp5l* pre-mRNA within the mouse cortex, although with around 3-fold higher levels of Sam68 (Figure 1B). Both Sam68 and SLM2 proteins bound to *Stxbp5l* RNA probes *in vitro* in FP experiments (Table 1). Analysis of physiological splicing patterns in the mouse cortex (Figure 1D) and other brain regions (quantitative data for *Stxbp5l* splicing patterns in the cortex and other mouse brain regions are shown in Supplementary Figure S1) showed that splicing of this 171 nucleotide *Stxbp5l* exon responded to knockout of either *Slm2* and *Sam68*. Hence, *Stxbp5l* is a joint splicing target for both SLM2 and Sam68, similar to the *Neurexin1* and *Neurexin3* AS4 exons, and unlike *Neurexin2*. Note an additional upstream 93 nucleotide exon is annotated in some mRNAs but not in Refseq, and we could detect some inclusion of this additional exon in the brain (to give the +93 band) but splicing of this did not respond to deletion of either *Sam68* or *Slm2*.

### The STAR domain of SLM2 establishes substrate specificity for the *Neurexin2* AS4 exon

**Figure 2.**
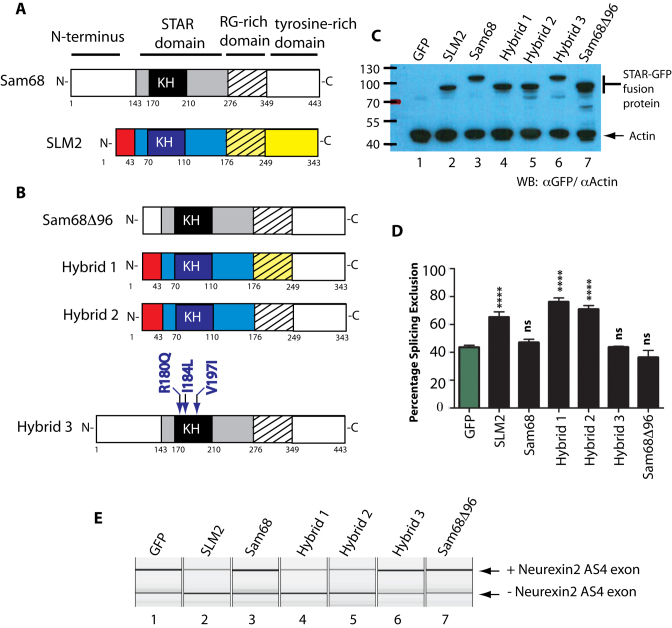
The STAR domain of SLM2 establishes substrate specificity for the Neurexin2 AS4 exon but not through amino acids involved in RNA–protein contacts. (**A**) Modular structures of SAM68 and SLM2 protein, showing the important domains above, and amino acid residue positions below each protein (1–343 for SLM2, and 1–443 for SAM68). (**B**) Variant SAM68 and SLM2 proteins used in splicing analyses, with SLM2-derived domains coloured as in part (A) to distinguish them from their corresponding SAM68-derived domains (in black and white). (**C**) Western blot showing relative expression of the different SLM2 and SAM68-GFP fusion proteins relative to actin. (**D**) Bar chart and (**E**) example capillary gel electrophoretogram showing levels of splicing of a *Neurexin2* AS4 exon from a co-transfected minigene (6) measured after expression of either a GFP fusion protein (black bars) or GFP only (green bar). Statistical significances were analysed using Graphpad and are indicated as **** (*P* < 0.0001), or ns (not significant).

We carried out a series of experiments to identify the region of SLM2 protein responsible for its differential splicing activity. SLM2 and Sam68 proteins have similar modular designs, although Sam68 protein is longer because of an additional 96 amino acid sequence at its N-terminus (Figure 2A). We first tested if the presence of this additional 96 amino acid sequence at the N-terminus was sufficient to prevent skipping of *Neurexin2* AS4 being induced by Sam68. Deletion of these 96 amino acids produced a shorter Sam68 protein (Sam68Δ96) that displayed a similar molecular weight to SLM2 and was efficiently expressed in HEK293 cells (Figure 2B and C). However, when Sam68Δ96 was co-expressed with a *Neurexin2* minigene, it was unable to induce skipping of the AS4 exon (Figure 2D and E), hence ruling out these 96 amino acids as a contributory factor to differential regulation of *Neurexin2* AS4 splicing.

To identify the domain in SLM2 that enables skipping of the *Neurexin2* AS4 exon, we carried out domain swap experiments, in which we replaced either the tyrosine-rich region of SLM2 (hybrid protein 1), or both the tyrosine rich region and RG-rich regions of SLM2 (hybrid protein 2), with their equivalent regions from Sam68 protein (Figure 2B). After co-transfection with the *Neurexin2* minigene (6), both of these hybrid SLM2 proteins were able to induce skipping of the AS4 exon as well as wild type SLM2 protein (Figure 2D and E).

The above results indicated that a protein region including the SLM2 STAR domain is responsible for establishing differential splicing repression of the *Neurexin2* AS4 exon between SLM2 and Sam68. The STAR domain of SLM2 and Sam68 is highly conserved and provides both a protein–RNA and protein–protein interaction interface (15,24). However, three amino acid positions show differences in RNA–protein contact between SLM2 and Sam68: these are arginine 180 in Sam68 which is substituted by glutamine in SLM2, isoleucine 184 which is a leucine in SLM2, and valine 197 which is isoleucine in SLM2 (15). To test if these variant protein–RNA contacts contribute to differential splicing regulation of *Neurexin2* AS4 via establishing subtle differences in RNA–protein contact, we constructed a version of Sam68 (called hybrid 3) in which the three amino acids were converted to those present in SLM2. Hybrid 3 protein was unable to induce skipping of *Neurexin2* AS4 in minigene co-transfection experiments, showing that the amino acids with variant RNA–protein contacts between Sam68 and SLM2 do not mediate the different splicing activity of these two splicing regulators on the *Neurexin2* AS4 exon (Figure 2D and E).

### A different intronic distribution of SLM2/SAM68 RNA binding sites flank the *Neurexin2* AS4 compared with both the *Neurexin1* and *Neurexin3* AS4 exons

**Figure 3.**
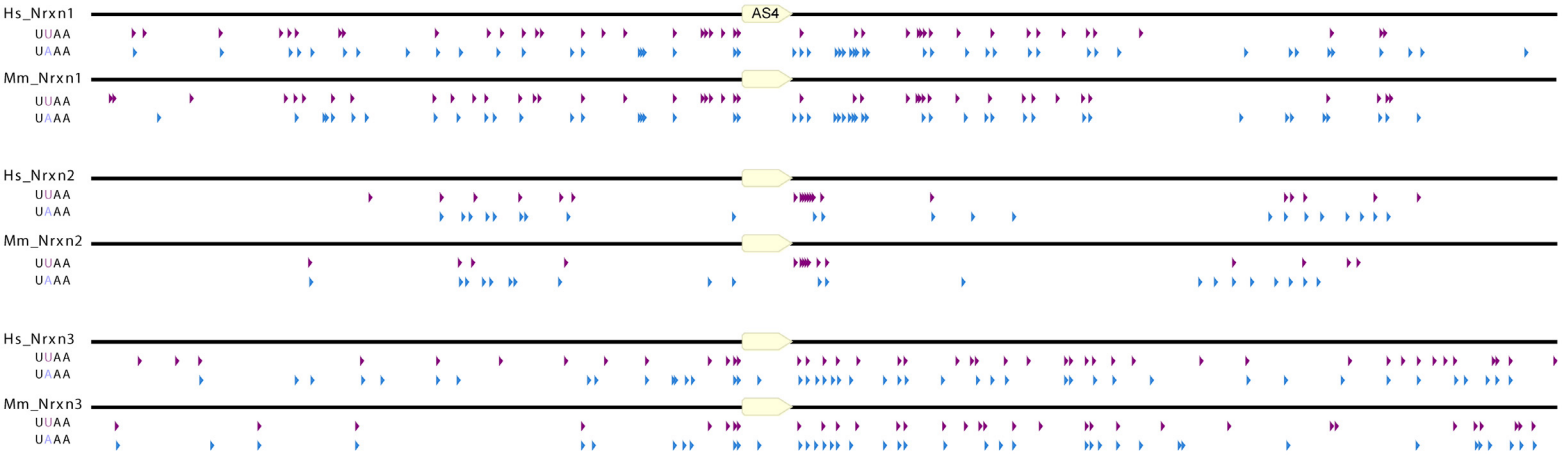
Genomic distribution of UWAA repeats bound by SLM2 and SAM68 proteins in the upstream and downstream introns flanking the *Neurexin1–3* gene AS4 exons. The pattern of UWAA motifs across the entire flanking intron sequences upstream and downstream of the AS4 exon (yellow box) are shown. SLM2 and SAM68 proteins both bind RNA as dimers to individual UWAA motifs separated by >15 nucleotides. Individual UWAA motifs are differentiated as purple (UUAA) and blue (UAAA) arrowheads. Abbreviations used are Hs: *Homo sapiens*; and Mm: *Mus musculus*.

Taken together, the above data suggested that different protein–RNA binding abilities between SLM2 and Sam68 do not differentiate the splicing specificity of these proteins on the *Neurexin2* pre-mRNA. Thus, we asked if sequence elements in the *Neurexin2* pre-mRNA itself could explain this differential effect. Since earlier genome comparisons had only examined intronic binding site patterns within the first 200 nucleotides downstream of each *Neurexin1–3* AS4 exon (6), we further mapped the entire flanking intronic sequences upstream and downstream of each AS4 exon for the presence of Sam68/SLM2 binding sites (Figure 3). The resulting human and mouse maps showed quite different distributions of Sam68/SLM2 RNA binding UWAA repeats flanking the *Neurexin2* AS4 exons, compared to the equivalent regions in the *Neurexin1* and *Neurexin3* genes.

A striking feature in SLM2/Sam68 binding site distributions was their particularly low overall occurrence flanking the *Neurexin2* AS4 exon (with the exception of the 51 nucleotide cluster). In contrast, UWAA repeat sequences were both more frequent and symmetrically distributed in the intronic regions flanking the mouse and human *Neurexin1* AS4 exons (Figure 3). There were also multiple and evenly distributed UWAA repeat sequences immediately flanking the mouse *Neurexin3* AS4 exon, although UWAA motifs were scarcer towards the more distant reaches of the upstream *Neurexin3* intron. We also observed a high density of UWAA motifs around the *Stxbp5l* alternative exon in both the mouse and human genomes, consistent with regulation by both SLM2 and Sam68 (Figure 1B) (Supplementary Figure S2).

These general patterns of SLM2/Sam68 binding sites flanking the *Neurexin2* AS4 exons are conserved across different species’ genomes, and particularly within mammals (Supplementary Figure S3). Thus within each mammalian genome sequence examined (humans, mice, Tasmanian devils, and Tammar wallabies; abbreviated Hs, Mm, Sh and Me respectively in Supplementary Figure S3), there is a tight cluster of UWAA binding sites in the intron immediately downstream of the *Neurexin2* AS4 yet relatively few other exon-proximal binding sites either upstream or downstream. Also, within each of these species, the *Neurexin1* and *Neurexin3* AS4 exons have much more extensive flanking distributions of UWAA repeats (particularly downstream repeats in the *Neurexin3* gene).

### Doubling the number of upstream or downstream SLM2/SAM68 binding site clusters enables skipping of the *Neurexin2* AS4 exon by both SAM68 and SLM2

**Figure 4.**
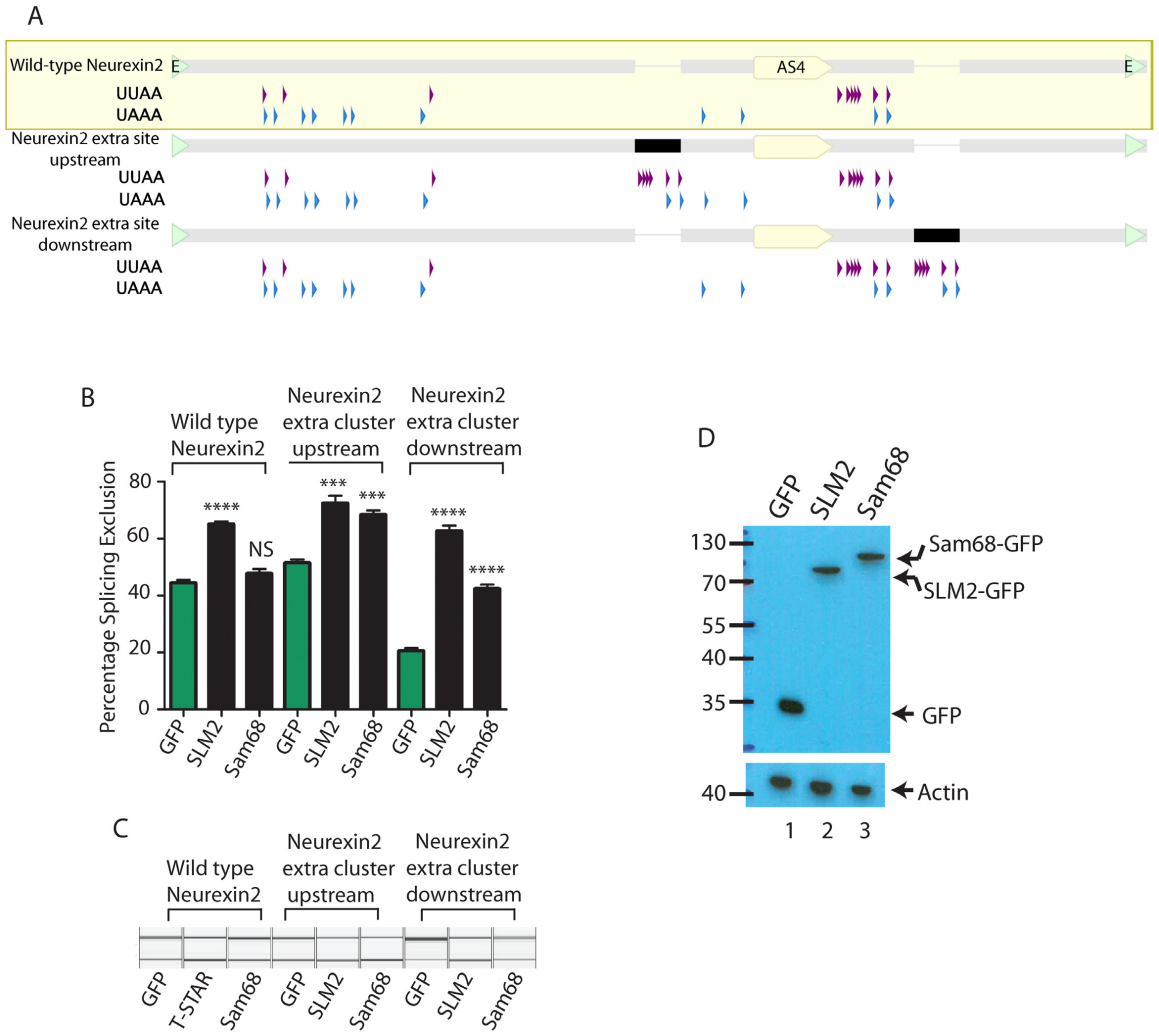
Increasing the density of UWAA repeats makes the *Neurexin2* AS4 exon respond to SAM68 as well as SLM2. (**A**) Distribution of UWAA repeats in the wild type *Neurexin2* AS4 minigene, and two engineered versions of this minigene that have an additional cluster placed downstream (intron position +112) or upstream (intron position -100) relative to the AS4 exon. The position of the *Neurexin2* AS4 exon is indicated as a yellow pointed bar within the wild type minigene and for each mutant. The position of the extra 51 nucleotide SLM2 response sequence in each new construct is shown as a black bar (or a thinner line where these are absent). The edges of the minigene insert, where they are inserted into the exon trap vector pXJ41 are shown as green arrowheads. (**B**) Percentage Splicing Exclusion of the *Neurexin2* AS4 exon after co-expression of the three minigenes with GFP, a SLM2-GFP fusion protein, or a SAM68-GFP fusion protein. (**C**) Representative capillary gel electrophoretograms showing splicing control of the wild type or engineered versions of the *Neurexin2* minigene. (**D**) Western blot showing expression levels of proteins in (B). On the bar chart, statistical significance was addressed using *t* tests. ****P < 0.0001; ***P < 0.001; NS, not significant. Error bars show standard error of the mean.

We engineered two new versions of the *Neurexin2* AS4 minigene (6) to test whether reduced binding site number is an important parameter in determining specific splicing repression by SLM2 compared to Sam68 (Figure 4A). Both these new minigenes were ‘wild type’ in that they contained the 51 nucleotide cluster of SLM2/Sam68 binding sites in its normal position immediately downstream of the AS4 exon. However, each new minigene contained an second additional copy of this 51 nucleotide cluster, placed either upstream or further downstream of the *Neurexin2* AS4 exon, and so recapitulates the distributions of binding sites found flanking the *Neurexin1* and *Neurexin3* AS4 exons. These new minigenes and the original wild type minigene were transfected into HEK293 cells, and the splicing repressive activity of SLM2 and Sam68 were measured by the absolute change in *Neurexin2* AS4 percentage splicing exclusion.

Strikingly, the extra 51 nucleotide cluster in both of these two new *Neurexin2* AS4 minigenes enabled efficient AS4 exon skipping by both Sam68 and SLM2 (Figure 4B and C). Hence, doubling the number of RNA binding sites, in an arrangement either both upstream and downstream, or just downstream, of the *Neurexin2* AS4 exon, is sufficient to confer responsiveness to Sam68 as well as SLM1. Of these two new minigenes, the version with the combination of 51 nucleotide binding site clusters located both downstream and upstream of the *Neurexin2* AS4 exon had the strongest effect on splicing control by Sam68, with no significant difference between the two STAR proteins (both of which are expressed at similar levels, Figure 4D) in measured splicing repression (*P* = 0.2285).

### The endogenous downstream position of the SLM2/Sam68 binding cluster does not enable the differential response of *Neurexin2* AS4 to SLM2

Although insertion of the extra 51 nucleotide element into the downstream site enabled efficient splicing control by both SAM68 and SLM2, this arrangement also decreased the background level of splicing exclusion of the Neurexin2 AS4 exon from ∼40% to ∼20% (Figure 4B and C), with SLM2 giving a stronger splicing repression than SAM68 (∼20% difference, *P* = 0.0001). We thus tested if the position of the 51 nucleotide SLM2/Sam68-binding site cluster downstream of the *Neurexin2* AS4 exon contributes to differential splicing control by SLM2.

**Figure 5.**
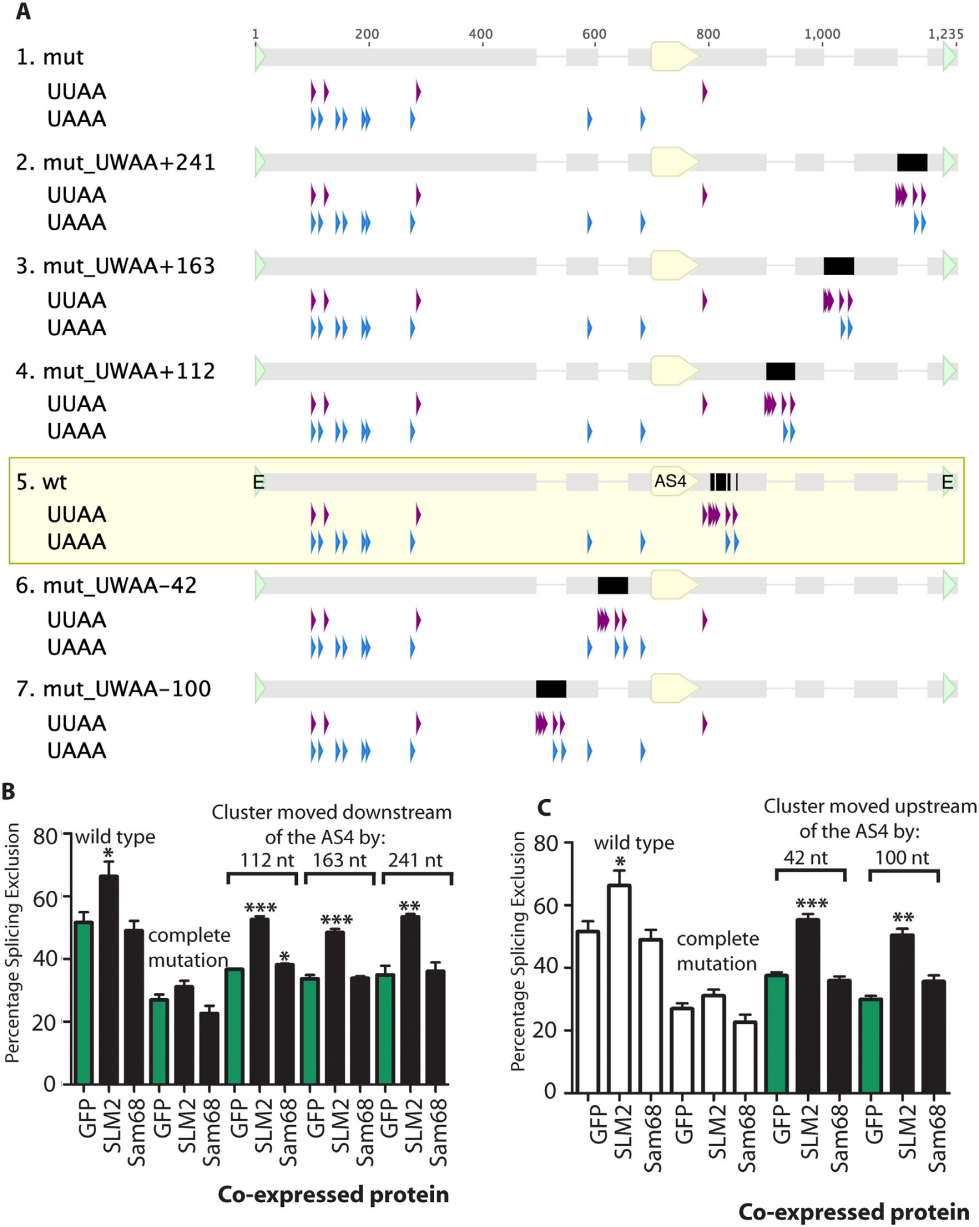
The SLM2 response element controlling the *Neurexin2* AS4 exon can operate from both upstream and further downstream of the target exon. (**A**) UUAA and UAAA sequence contents of wild type *Neurexin2* AS4 minigene and minigenes that have the binding site cluster moved to various intronic positions upstream or downstream of the AS4 exon. The minigene insert sequence cloned into the pXJ41 exon trap vector is shown, with nucleotide numbering from the first to last nucleotide of this inserted sequence. The position of the *Neurexin2* AS4 exon is indicated as a yellow pointed bar under the wild type and each mutant. The position of the 51 nucleotide SLM2 response sequence in each construct is shown as a black bar. (**B**) Effect of moving the UWAA cluster downstream of the *Neurexin2* AS4 exon within the minigene construct, shown a bar chart. (**C**) Effect of moving the 51 nucleotide UWAA cluster upstream of the *Neurexin2* AS4 exon on exon skipping, shown as a bar chart (data from three biological replicates). Statistical significance was addressed using t tests, and is shown on the bar charts as: ***P < 0.001; **P < 0.01; *P < 0.05. Error bars show standard error of the mean. Data previously shown in earlier panels is shown as white bars.

We made a new set of minigenes within a background in which the endogenous 51 nucleotide cluster had already been mutated to block splicing regulation by SLM2 (this starting minigene is called the complete mutation (6)). An entire 51 nucleotide SLM2/Sam68 binding site cluster was inserted at sites 112, 163 and 241 nucleotides downstream of the 5΄ splice site of the *Neurexin2* AS4 alternative exon (Figure 5A). In each case the 51 nucleotide cluster of Sam68/SLM2 RNA binding sites enabled splicing repression by co-transfected SLM2 protein just as well as when it was in the wild type location immediately downstream of the regulated exon (Figure 5B).

Next, we tested whether switching the 51 nucleotide response element into the upstream flanking intron could also restore sensitivity to SLM2. In order to avoid possible insertions into either the branchpoint or the polypyrimidine tract that are needed for splicing inclusion of the *Neurexin2* AS4 exon, we selected regions for insertion of this element 42 nucleotides and 100 nucleotides upstream of the *Neurexin2* AS4 exon (25). Both upstream insertions had no effect on AS4 splicing inclusion when the minigene was co-transfected with GFP (Figure 5C), showing that no essential splicing signals had been destroyed. However, for both these constructs, co-transfection of SLM2 protein still strongly promoted *Neurexin2* AS4 exon skipping (Figure 5C). But, in each case, splicing repression was only exerted by SLM2 and not by Sam68, indicating that SLM2 can operate in splicing control from different positions relative to the regulated exon, and that the original immediately downstream position is also not a factor that prevents Sam68-mediated splicing control of this AS4 exon.

### SLM2 can efficiently regulate the *Neurexin2* AS4 exon even when it is further depleted of UWAA STAR protein binding sites

The above experiments indicate that a difference in binding site density flanking the regulated exon enables splicing regulation of the *Neurexin2* AS4 exon by SLM2 and not Sam68. The 51 nucleotide element itself contains a number of SLM2/Sam68 RNA binding sites. To test how many individual Sam68/SLM2 binding sites are sufficient to enable splicing repression of *Neurexin2* AS4 by SLM2, we further dissected the 51 nucleotide sequence within its normal location.

**Figure 6.**
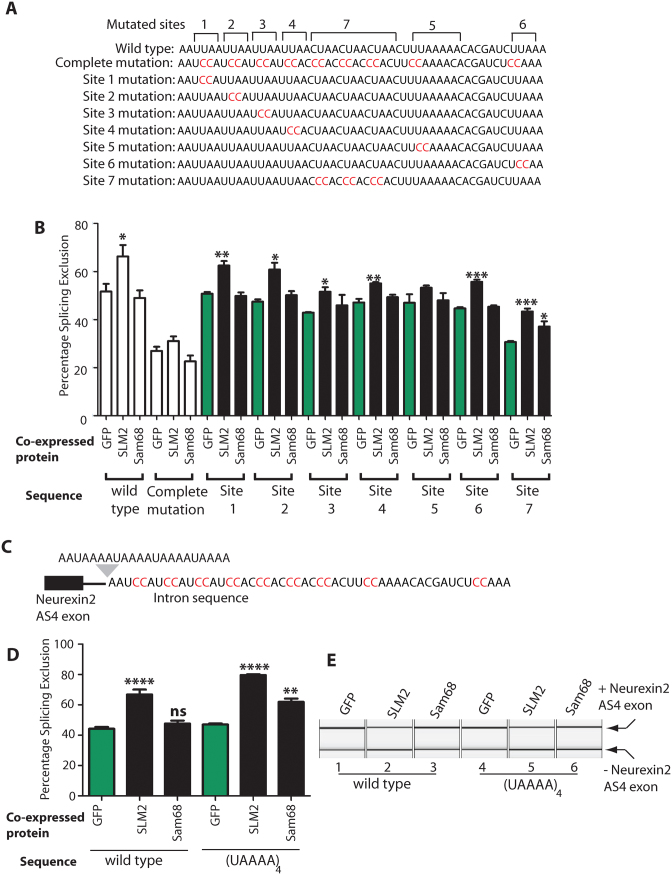
Dissection of the 51 nucleotide SLM2 response element downstream of the Neurexin2 AS4 exon. (**A**) Sequence repeat composition of the 51 nucleotide cluster of Sam68/SLM2 binding sites downstream of the *Neurexin2* AS4 exon (6), with the individual tested sequence elements in mutants 1–7 shown underneath. In each case, the mutated nucleotides are shown in red, and wild type nucleotides are black. (**B**) Effects on splicing exclusion of the *Neurexin2* AS4 exon, after mutating individual components of this repeat sequence shown as a bar chart. (**C**) Minigene structure after the endogenous 51 nucleotide SLM2 response element is mutated, and a (UAAAA)_4_ sequence inserted immediately upstream of this mutated sequence. (**D**) Bar chart and (**E**) example capillary gel electrophoretogram showing splicing response of the *Neurexin2* AS4 exon after the 51 nucleotide SLM2 response element is substituted with a (UAAAA)_4_ sequence. Data shown in all the bar charts are averages from at least three biological replicates, with splicing levels in cells transfected with GFP data shaded green and splicing levels in cells transfected with SAM68 and SLM2 shaded black. Statistical significance was addressed using t tests, and is indicated on the bar chart as ****P < 0.0001; ***P < 0.001; **P < 0.01; *P < 0.05. Error bars show standard error of the mean. Data previously shown in earlier panels is shown as white bars.

The 51 nucleotide cluster of SLM2/Sam68 binding sites downstream of the *Neurexin2* AS4 exon contains three distinct sequence elements (Figure 6A): four UUAA repeats (sites 1–4 in Figure 6A); directly followed by three CUAA repeats (site 7 in Figure 6A); followed by a more extended AU-rich element (containing UUAAAAA and UUAAA, which are sites 5 and 6 respectively in Figure 6A). Individual mutation of each of the UUAA repeats led to decreased splicing repression by SLM2, but in each case splicing repression was still statistically significant compared with the same minigene co-transfected with GFP (sites 1–4, Figure 6B. In particular mutation of sites 3 and 4 resulted in a relatively modest but still significant PSE change). Similarly, the *Neurexin2* AS4 exon was still repressed by SLM2 after mutation of the UUAAA sequence (site 6, Figure 6B). Following each change in the 51 nucleotide element, splicing repression was only via SLM2 protein and not Sam68. The strongest effects on SLM2-mediated exon skipping was caused by individual mutation of the UAAAA sequence (site 5, Figure 6B), after which the *Neurexin2* AS4 exon was no longer significantly repressed by SLM2. Individual mutation of the CUAA repeat region (site 7) did not prevent splicing repression by SLM2, but reduced the overall levels of splicing exclusion from 52% to 31% (site 7 in Figure 6B) in cells co-transfected with GFP, suggesting that site 7 may correspond to a normally strong repressive sequence that is disrupted by the mutation (or alternatively the mutation may create an enhancer).

The strongest effect on exon skipping in the above experiments was thus mutation of the UAAAA sequence (position 5 in Figure 6A). FP analysis of a (UAAAA)_4_ sequence confirmed that this sequence efficiently binds to the STAR domains of SLM2 and Sam68 proteins in vitro, with a similar dissociation constant to the full 51 nucleotide *Neurexin2* response element (Table 1). To test if an increased number of UAAAA sites would be sufficient by itself to enable *Neurexin2* AS4 splicing control by Sam68 as well as SLM2, we constructed a minigene that contained four consecutive repeats of this sequence, (UAAAA)_4_, to replace the endogenous single copy of the 51 nucleotide sequence (previously removed by the complete mutation). The AS4 exon from this (UAAAA)_4_ minigene construct was included within mRNAs at similar levels to the wild type control when co-transfected with GFP (Figure 6C). Insertion of the (UAAAA)_4_ sequence downstream of the AS4 exon enabled very efficient splicing repression by co-expressed SLM2 protein. In fact, the splicing response of the (UAAAA)_4_ minigene to SLM2 co-expression was stronger than the wild type minigene (∼18% difference, *P* = 0.0332). Furthermore, the addition of these four downstream UAAAA repeats were sufficient to place the *Neurexin2* AS4 exon under joint splicing control by Sam68 protein, although this was not as strong as for SLM2. These experiments confirm that the UAAAA sequence (position 5 in Figure 6A) is the major determinant of splicing repression by SLM2 protein, and that Sam68 requires increased density of this site (in this case four copies instead of one) to cause exon skipping.

## DISCUSSION

**Figure 7.**
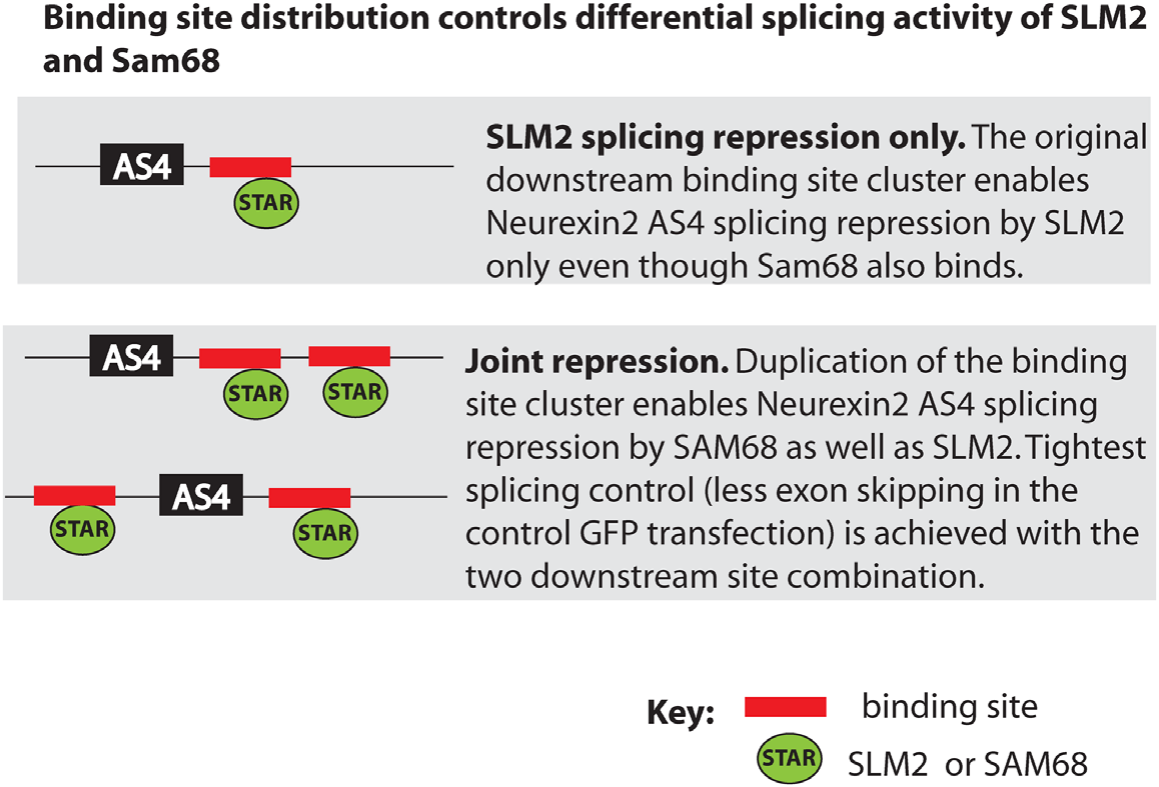
Binding site density controls differential splicing activity of SLM2 and Sam68 on the *Neurexin2* AS4 exon. This model proposes that the single binding site cluster downstream of the *Neurexin2* AS4 exon binds both Sam68 and SLM2 proteins, but only SLM2 is proficient to cause AS4 skipping. Duplication of this binding site cluster enables joint regulation by both SLM2 and Sam68.

Here, we propose differential splicing regulation by SLM2 and Sam68 involves the density of RNA–protein binding sites that flank target exons within the *Neurexin* pre-mRNAs, rather than the actual core binding site sequence *per se*. Notably, this is the first study that has examined the basis of interchangeability between two STAR protein paralogs *in vivo*. Our data support a model (Figure 7) where *Neurexin2* AS4 has a reduced density of flanking Sam68/SLM2 binding sites that can only support splicing control by SLM2. Three lines of evidence led to this model. Firstly, CLIP experiments (Figure 1B) monitoring *in vivo* binding within the mouse cortex and *in vitro* RNA binding assays (Table 1) indicate no apparent differences in Sam68 and SLM2 protein binding to the *Neurexin2* pre-mRNA, even though only SLM2 protein and not Sam68 can repress splicing of this exon in the mouse brain or in transfected cells. Secondly, based on the recent atomic-level characterization of SLM2 and Sam68 RNA–protein contacts (15), changing the three Sam68 amino acid residues within the STAR domain with different RNA–protein contacts into their equivalent amino acid residues in SLM2 (which should in effect turn Sam68 into SLM2 in terms of RNA–protein contact) was not sufficient to enable splicing regulation of *Neurexin2* AS4 by Sam68 (Figure 2) (15). Thirdly, genome analysis indicated much fewer intronic Sam68/SLM2 binding sites flank the *Neurexin2* AS4 exon, compared to *Neurexin1* and *Neurexin3* which are under joint control by SAM68 protein as well (Figure 3). Similar patterns of SLM2/Sam68 binding sites flanking the *Neurexin1–3* AS4 exons are also conserved across all mammals, consistent with them being functionally important (Supplementary Figure S3). Thus, all mammalian *Neurexin2* AS4 exons have fewer flanking UWAA repeat sequences and these are mainly concentrated within a single downstream cluster, and in each case are more similar to each other in UWAA distribution across species than to the corresponding pattern for the *Neurexin1* or *Neurexin3* gene within the same species.

Experimentally confirming a prediction of this model, duplication of the entire intronic 51 nucleotide element downstream of the *Neurexin* 2 AS4 exon was sufficient to enable joint splicing regulation by Sam68 as well as SLM2 (Figure 4B and C). We predict that a finite amount of SLM2 protein bound to the *Neurexin2* pre-mRNA is more proficient in its ability to repress *Neurexin2* AS4 splicing than a finite amount of bound Sam68 protein, and that increased levels of Sam68 binding enabled by binding cluster duplication enables joint splicing regulation despite this lower proficiency. Binding site cluster duplication also made the introns flanking the *Neurexin2* AS4 exon more closely resemble those that flank the *Neurexin1* and *Neurexin3* AS4 exons, thus explaining at a genomic level why *Neurexin1* AS4 and *Neurexin3* AS4 exons are under joint Sam68/SLM2 splicing control. Also consistent with this prediction, we observed that Sam68 can modulate alternative splicing of another physiological SLM2 target exon in the *Stxbp5l* gene (Figure 1C) that also contains higher densities of flanking UWAA binding sites than the *Neurexin2* AS4 exon (Supplementary Figure S2). *Stxbp5l* pre-mRNA also bound higher levels of Sam68 within the mouse cortex, possibly because of higher endogenous expression levels of Sam68 relative to SLM2. Previous work has shown *Sgce* exon 8, which is also controlled in the mouse brain by Sam68, contains many intronic UAAA sequences both upstream and downstream of the exon, and Sam68 binding in this case also causes exon exclusion (26,27).

Interestingly, although SLM2/Sam68 binding site clusters can mediate *Neurexin2* AS4 splicing control from both upstream and downstream intronic positions, the highest fold splicing switch in response to SLM2 and Sam68 co-expression was achieved with the binding site cluster duplicated downstream of the AS4 exon. An immediately downstream position for this cluster of SLM2/Sam68 binding sites has also been conserved for the endogenous *Neurexin2* AS4 exon within each of the genomes we analysed, although our minigene data also indicate that this single cluster can mediate exclusive SLM2-splicing control from either upstream or downstream intronic positions. A duplicated downstream intronic binding site cluster did however give a tighter pattern of control, with less exon skipping after co-transfection with GFP. While specific RNA–protein contacts did not detectably contribute to differential splicing regulation of *Neurexin2* AS4 by SLM2 and Sam68, STAR domains are both RNA and protein interaction interfaces, mediating their ability to homodimerize and heterodimerize (15,28). As well as RNA binding, protein dimerisation is essential for the splicing repressive activity of both SLM2 and Sam68 (15,16). SLM2 and Sam68 heterodimerise, and co-transfection of Sam68 with SLM2 protein inhibits the activity of SLM2 in splicing control of *Neurexin2* AS4, presumably through inducing formation of a heterodimer that is no longer sufficient to mediate skipping of this exon (6).

More generally, many splicing regulator proteins exist in multi-protein families. Multi-protein families may enable individual protein members to have evolved different patterns of expression, thus providing a mechanism to vary local concentrations of splicing regulator proteins like Sam68 (fairly ubiquitously expressed) and SLM2 (mainly expressed in the testis and brain). The existence of multi-protein families could also enable individual protein members of each family to specialise in splicing control of different targets by evolving different RNA binding specificities, although the STAR family members Sam68 and SLM2 have been puzzling in sometimes selecting different functional targets while having indistinguishable RNA binding sites (15). Our model presented here, that splice site density within target pre-mRNAs influences splicing responses to Sam68 and SLM2, might also apply to other families of splicing factors that have indistinguishable RNA target sites but that control different subsets of splicing targets.

## Supplementary Material

Supplementary Data
