# Supplementary material for: Binding site density enables paralog-specific activity of SLM2 and Sam68 proteins in Neurexin2 AS4 splicing control
Source: Nucleic Acids Res. 2016 Dec 19;45(7):4120–30. doi: 10.1093/nar/gkw1277 (PMC5397175; doi:10.1093/nar/gkw1277)
Supplement: Supplementary Data [file gkw1277_Supp.zip › nar-03728-a-2015-File009.pdf]

SUPPLEMENTARY FIGURES

Supplementary File 1. Sequences of oligonucleotides used in this study.

Supplementary Figure S1 Analysis of *Stxbp5l* alternative splicing patterns in different brain regions from wild type, *SLM2* and *Sam68* null mice. (A,B) Percentage splicing exclusion of the 171 nucleotide *Stxbp5l* variable exon in different brain regions of wild type, homozygous *Slm2* (A) and *Sam68* (B) knockout mice, measured using RT-PCR and capillary or agarose gel electrophoresis, respectively. Data shown in the bar chart are averages from three biological replicates. On the bar chart, statistical significance was addressed using t tests: *** $p<0.001$; ** $p<0.01$; * $p<0.05$; ns, not significant. Error bars show standard deviation of the mean.

Supplementary Figure S2. Structure of the *Stxbp5l* 171 nucleotide alternative exon and its flanking intron sequence. The exon sequence is shown as a yellow box in each case, and UUAA and UAAA motifs as red and blue triangles respectively. The two downstream motif clusters that were analysed for binding affinity to SLM2 using FP in Table 1 are labelled 1 and 2.

Supplementary Figure S3. Phylogenetic analysis shows that UWAA repeats are conserved upstream and downstream of the *Neurexin AS4* exons in amniotes. The pattern of UWAA motifs across the entire upstream and downstream flanking intron sequences is shown, with individual UWAA motifs indicated as a purple arrowhead. Abbreviations used are Hs (Homo sapiens); Mm (Mus musculus); Sh (Sarcophilus harrisii); Me (Macropus eugenii); Oa (Ornothorhynchus anatinus); Ps (Pelodiscus sinensis); Am (Alligator mississippiensis); Xt (Xenopus tropicalis); Dr (Danio rerio).

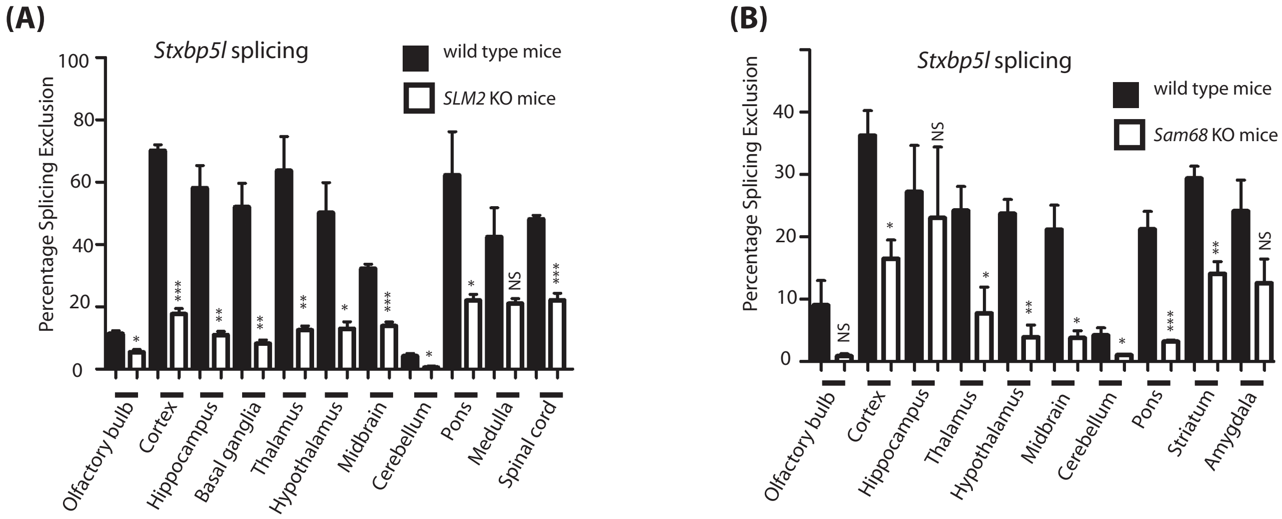
(A)
Stxbp5l splicing
wild type mice
SLM2 KO mice
Percentage Splicing Exclusion
100
80
60
40
20
0
Olfactory bulb
Cortex
Hippocampus
Basal ganglia
Thalamus
Hypothalamus
Midbrain
Cerebellum
Pons
Medulla
Spinal cord
NS
(B)
Stxbp5l splicing
wild type mice
Sam68 KO mice
Percentage Splicing Exclusion
40
30
20
10
0
Olfactory bulb
Cortex
Hippocampus
Thalamus
Hypothalamus
Midbrain
Cerebellum
Pons
Striatum
Amygdala
NS

Figure S1

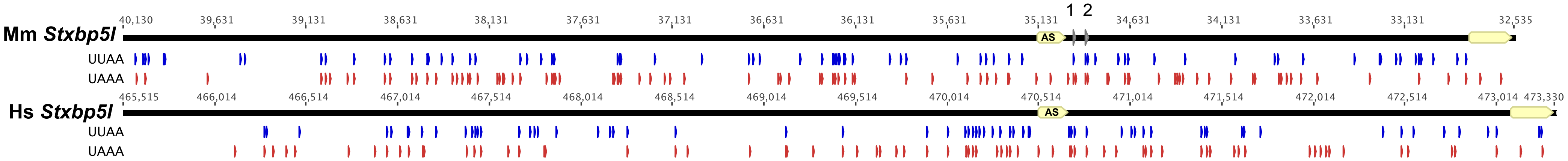

Structure of mouse (Mm) and human (Hs) *Stxbp5l* 171 nucleotide alternative exons and flanking intron sequences.

Exon sequences are shown as yellow boxes, and UUAA and UAAA motifs as red and blue triangles respectively. The two downstream motif clusters that were analysed for binding affinity to SLM2 using FP in Table 1 are labelled 1 and 2. Numbering corresponds to genomic sequences (mouse MGI:2443815, human HGNC:19665). AS: alternatively spliced.

Figure S2

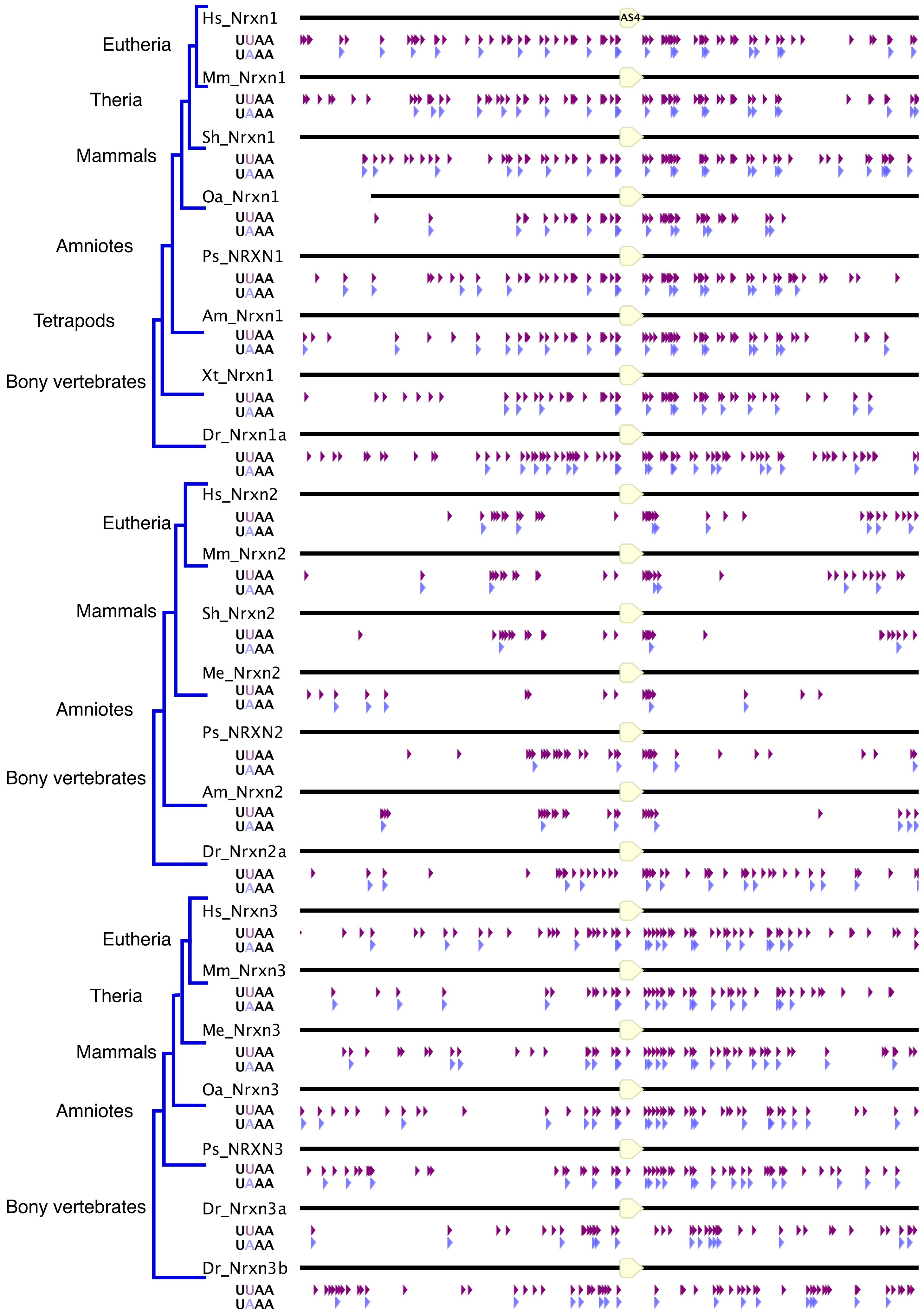
AS4
Eutheria
Theria
Mammals
Amniotes
Tetrapods
Bony vertebrates
Hs_Nrxn1
Mm_Nrxn1
Sh_Nrxn1
Oa_Nrxn1
Ps_NRXN1
Am_Nrxn1
Xt_Nrxn1
Dr_Nrxn1a
Hs_Nrxn2
Mm_Nrxn2
Sh_Nrxn2
Me_Nrxn2
Ps_NRXN2
Am_Nrxn2
Dr_Nrxn2a
Hs_Nrxn3
Mm_Nrxn3
Me_Nrxn3
Oa_Nrxn3
Ps_NRXN3
Dr_Nrxn3a
Dr_Nrxn3b
UUAA
UAAA

Figure S3
