# Supplementary material for: Binding site density enables paralog-specific activity of SLM2 and Sam68 proteins in Neurexin2 AS4 splicing control
Source: Nucleic Acids Res. 2016 Dec 19;45(7):4120–30. doi: 10.1093/nar/gkw1277 (PMC5397175; doi:10.1093/nar/gkw1277)
Supplement: Supplementary Data [file gkw1277_Supp.zip › nar-03728-a-2015-File010.docx]

**Primer List**

| **Primer** | **Sequence** |
| --- | --- |
| pXJB1vector primer | GCTCCGGATCGATCCTGAGAACT |
| pXJRTF vector primer | GCTGCAATAAACAAGTTCTGCT |
| *Nrxn2* Big Mutation F | AATCCATCCATCCATCCACCCACCCACCCACTTCCAAAACACGATCTCCAAGGTGCAGAGCTCTCTC |
| *Nrxn2* Big Mutation R | GGAGATCGTGTTTTGGAAGTGGGTGGGTGGGTGGATGGATGGATGGATTCTGGTTAATTACCTTTGTC |
| *Nrxn2* Mutant Site 1 F | AATCCATTAATTAATTAACTAACTAACTAACTTTAAAAACACGATCTTAAAAAGGTGCAGAGCTCTCTC |
| *Nrxn2* Mutant Site 1 R | TTTAAGATCGTGTTTTTAAAGTTAGTTAGTTAGTTAATTAATTAATGGATTCTGGTTAATTACCTTTGTC |
| *Nrxn2* Mutant Site 2 F | AATTAATCCATTAATTAACTAACTAACTAACTTTAAAAACACGATCTTAAAAAGGTGCAGAGCTCTCTC |
| *Nrxn2* Mutant Site 2 R | TTTAAGATCGTGTTTTTAAAGTTAGTTAGTTAGTTAATTAATGGATTAATTCTGGTTAATTACCTTTGTC |
| *Nrxn2* Mutant Site 3 F | AATTAATTAATCCATTAACTAACTAACTAACTTTAAAAACACGATCTTAAAAAGGTGCAGAGCTCTCTC |
| *Nrxn2* Mutant Site 3 R | TTTAAGATCGTGTTTTTAAAGTTAGTTAGTTAGTTAATGGATTAATTAATTCTGGTTAATTACCTTTGTC |
| *Nrxn2* Mutant Site 4 F | AATTAATTAATTAATCCACTAACTAACTAACTTTAAAAACACGATCTTAAAAAGGTGCAGAGCTCTCTC |
| *Nrxn2* Mutant Site 4 R | TTTAAGATCGTGTTTTTAAAGTTAGTTAGTTAGTGGATTAATTAATTAATTCTGGTTAATTACCTTTGTC |
| *Nrxn2* Mutant Site 5 F | AATTAATTAATTAATTAACTAACTAACTAACTTCCAAAACACGATCTTAAAAAGGTGCAGAGCTCTCTC |
| *Nrxn2* Mutant Site 5 R | TTTAAGATCGTGTTTTGGAAGTTAGTTAGTTAGTTAATTAATTAATTAATTCTGGTTAATTACCTTTGTC |
| *Nrxn2* Mutant Site 6 F | AATTAATTAATTAATTAACTAACTAACTAACTTTAAAAACACGATCTCCAAGGTGCAGAGCTCTCTC |
| *Nrxn2* Mutant Site 6 R | CTTGGAGATCGTGTTTTTAAAGTTAGTTAGTTAGTTAATTAATTAATTAATTCTGGTTAATTACCTTTGTC |
| *Nrxn2* Mutant Site 7 F | AATTAATTAATTAATTAACCCACCCACCCACTTTAAAAACACGATCTTAAAGGTGCAGAGCTCTCTC |
| *Nrxn2* Mutant Site 7 R | TTTAAGATCGTGTTTTTAAAGTGGGTGGGTGGGTTAATTAATTAATTAATTCTGGTTAATTACCTTTGTC |
| *Nrxn2* Mutant 1-4 F | AATCCATCCATCCATCCACTAACTAACTAACTT |
| *Nrxn2* Mutant 1-4 R | TGGATGGATGGATGGATTCTGGTTAATTACCTTTG |
| *Nrxn2* Mutant 145 F | ACTAACTAACTAACTTCCAAAACACGATCTTAAAGGTG |
| *Nrxn2* Mutant 145 R | CTTTAAGATCGTGTTTTGGAAGTTAGTTAGTTAGT |
| *Nrxn2* Mutant with sequence moved 112bp downstream F | AATTAATTAATTAATTAACTAACTAACTAACTTTAAAAACACGATCTTAAACCCTGCGTTCAGGCCGTTTG |
| *Nrxn2* Mutant with sequence moved 112bp downstream R | TTTAAGATCGTGTTTTTAAAGTTAGTTAGTTAGTTAATTAATTAATTAATTGGGCAGAAGGGAGCATCCTG |
| *Nrxn2* Mutant with sequence moved 163bp downstream | AATTAATTAATTAATTAACTAACTAACTAACTTTAAAAACACGATCTTAAAAAAGTCTTCCTAGAGTG |
| *Nrxn2* Mutant with sequence moved 163bp downstream F | TTTAAGATCGTGTTTTTAAAGTTAGTTAGTTAGTTAATTAATTAATTAATTCTCTTCCCTATTCCCAGC |
| *Nrxn2* Mutant with sequence moved 241bp downstream | AATTAATTAATTAATTAACTAACTAACTAACTTTAAAAACACGATCTTAAAGGAACACAGGCTAGAA |
| *Nrxn2* Mutant with sequence moved 241bp downstream R | TTTAAGATCGTGTTTTTAAAGTTAGTTAGTTAGTTAATTAATTAATTAATTCCTCCTACTGCAGAATT |
| *Nrxn2* Mutant 4xUAAAA F | AAGGTAATTAACCAGAATAAAATAAAATAAAATAAAACCCACCCACCCACTTC |
| *Nrxn2* Mutant 4xUAAAA R | GTGGGTTTTATTTTATTTTATTTTATTCTGGTTAATTACCTTTGTCG |
| Nrxn2 Ex20 FW | TAGATGAGTGGCTGCTCGAC |
| Nrxn2 Int21 REV | GGGAGAGAGCTCTGCACCTTT |
| Stxbp5l Ex22 FW | TCAGCTGCTTGCATGGAGATTT |
| Stxbp5l Int22 REV | TGTCTCCCAGCACCTGTTAC |
| Nrxn2 Ex19 FW | GTGCGCTTTACTCGAAGTGGTG |
| Nrxn2 Ex21 REV | CCCATTGTAGTAGAGGCCGGAC |
| Stxbp5l FW | CTGCAAGTCCCCCACCTCAG |
| Stxbp5l REV | ACAAACAGACAAGGGGAAACGG |
